# Genome sequences of six *Enterobacter* phages of the genus *Karamvirus*

**DOI:** 10.1128/mra.00606-26

**Published:** 2026-07-27

**Authors:** Tracey L. Peters, Min Tong, Yunxiu He, Caitlin D. Urick, Nino Mzhavia, Mikeljon P. Nikolich, Andrey A. Filippov

**Affiliations:** 1 Department of Animal, Veterinary and Food Sciences, College of Agricultural and Life Sciences, University of Idaho 5640, Moscow, Idaho, USA; 2 Wound Infections Department, Bacterial Diseases Branch, Center for Infectious Disease Research, Walter Reed Army Institute of Research 8394, Silver Spring, Maryland, USA; Queens College Department of Biology, Queens, New York, USA

**Keywords:** *Enterobacter cloacae*, drug resistance, phages, *Karamvirus*, whole genomes, virulent phages, phage therapy

## Abstract

Here, we describe the genomes of six *Enterobacter* bacteriophages (phages) of the genus *Karamvirus*. The range of genome length, GC content, and number of predicted protein-coding sequences were, respectively, 171,900–175,675 bp, 39.54–39.82%, and 298–316. Genomic analysis indicates that these phages have a lytic lifestyle and are suitable for therapeutic use.

## ANNOUNCEMENT

The *Enterobacter cloacae* complex is a group of nosocomial pathogens with prevalent multidrug-resistant (MDR) strains (1). Phages are promising therapeutics against MDR *E. cloacae* infections (2). Here, we report the genomes of six phages isolated on drug-resistant *E. cloacae* clinical isolates (Table 1).

**TABLE 1:** Genomic properties of six *E. cloacae* phages of the genus *Karamvirus*

Phage full name	Short name	Isolation date	Host strain	Raw read no.	Average read coverage	Genome length, bp	GC %	No. of CDSs^*a*^	tRNA gene nos.	BACPHLIP score	BioSample accession no.	SRA accession no.	GenBank accession no.
vB_Ecl466880-EEn2	EEn2	9/9/2019	MRSN 466880	223,776	293.4	172,366	39.75	307	17	100.00	SAMN55936664	SRR37356569	PZ086326
vB_Ecl476498-EEn8	EEn8	9/9/2019	MRSN 476498	750,914	626.0	171,912	39.82	304	19	98.75	SAMN55936665	SRR37356568	PZ086331
vB_Ecl420170-EEn25	EEn25	9/9/2019	MRSN 420170	1,344,772	1,135.2	171,900	39.70	310	19	95.00	SAMN55936666	SRR37356567	PZ086327
vB_Ecl420170-EEn26	EEn26	9/9/2019	MRSN 420170	826,822	678.1	175,675	39.64	314	19	97.42	SAMN55936667	SRR37356566	PZ086328
vB_Ecl506504-EEn31	EEn31	9/9/2019	MRSN 506504	1,287,634	1,066.1	172,035	39.54	298	16	85.00	SAMN55936668	SRR37356565	PZ086329
vB_Ecl32345-EEn35	EEn35	9/9/2019	MRSN 32345	978,322	773.8	173,697	39.75	316	19	97.50	SAMN55936669	SRR37356564	PZ086330

^*a*^ CDSs, protein-coding sequences.

The phages were isolated from sewage collected in Washington, DC (GPS coordinates: 38°49′09.2″ N, 77°01′16.0″ W), enriched in broth as described previously (3), and purified by three single-plaque isolations. Phage DNA was extracted from lysates with the QIAamp DNA Mini Kit (Qiagen, Germantown, MD, USA). Libraries were created using the KAPA HyperPlus Kit (Roche Diagnostics, Indianapolis, IN, USA) and sequenced on an Illumina MiSeq (Illumina, San Diego, CA, USA) with a 600-cycle MiSeq Reagent Kit v3 that produced 300-bp paired-end reads.

Paired-end reads (Table 1) were assessed for quality and trimmed using Fastp (4) v0.22.0. If required, reads were subsampled using seqtk (https://github.com/lh3/seqtk) v1.4 to achieve ~100× of the expected genome length. Trimmed reads were assembled with Unicycler (5) v0.5.0, and average coverage was calculated using BBMap (https://jgi.doe.gov/data-and-tools/bbtools/) v38.90 and SAMtools (6) v1.13. Genome termini were assessed by PhageTerm (7) v4.3. Phage lifestyle was predicted with BACPHLIP (8) v0.9.6. Annotation of protein and tRNA coding sequences (CDSs) was performed using the Pharokka pipeline (9–20) v1.6.0. Phage contigs were matched to their closest hits in the INPHARED database (19) using mash (20) v2.3. Default parameters were used for all tools.

**Fig 1:**
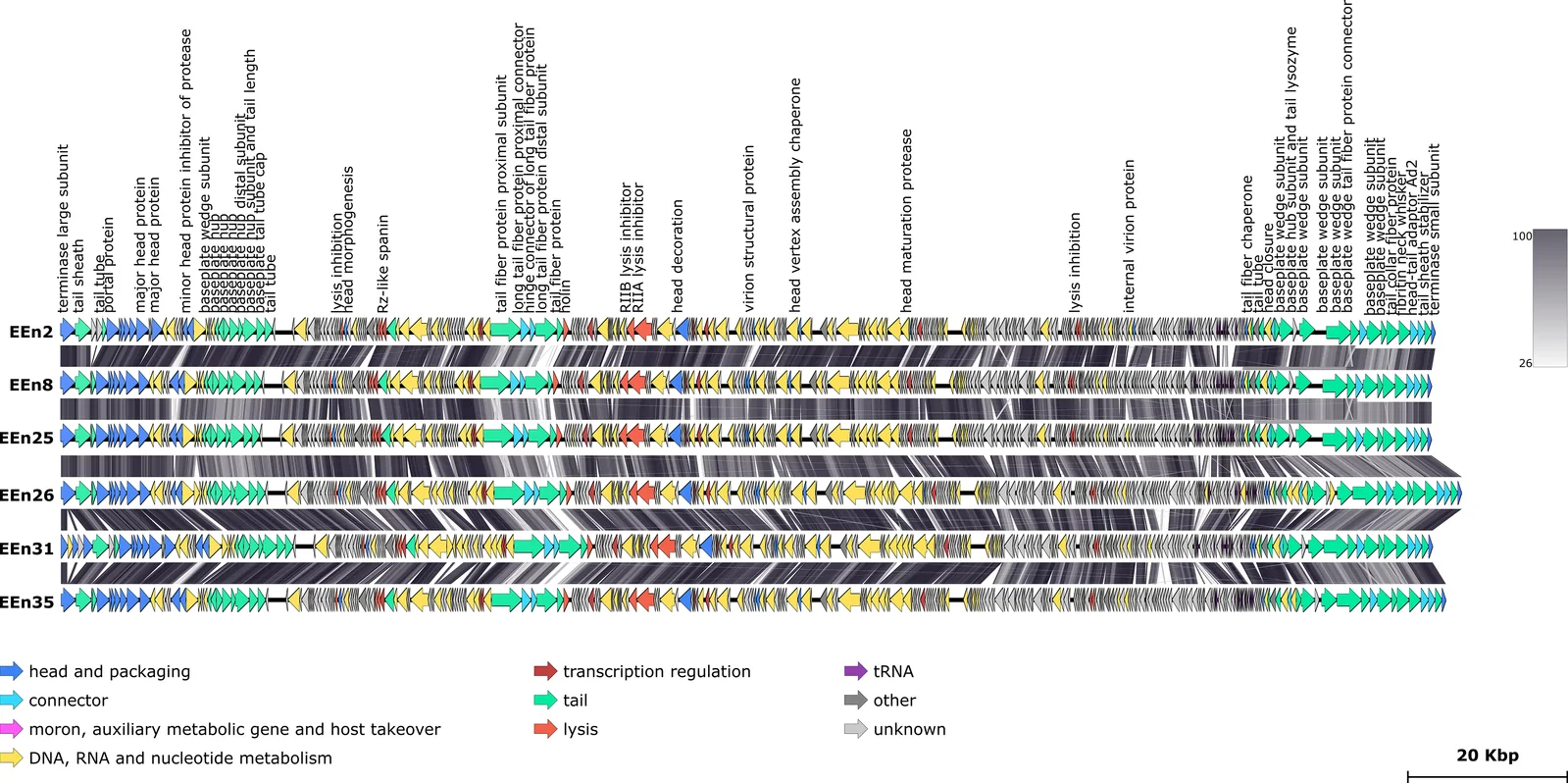
Genome map of six *Karamvirus* phages. The figure is based on Pharokka annotations, generated using GenoFig (28) v1.1.1 and edited in Inkscape (https://inkscape.org) v1.4.3.

Average read coverages for the six *Enterobacter* phage genomes varied in the range of 293–1,135×. The genome lengths, GC contents, numbers of CDSs, and tRNA genes fluctuated within 171,900–175,675 bp, 39.54–39.82%, 298–316, and 16–19, respectively (Table 1). Mash alignment (20) against the INPHARED database (19) classified these phages in the family *Straboviridae*, subfamily *Tevenvirinae*, genus *Karamvirus*. PhageTerm (7) analysis indicated that these phages have circularly permuted genomes with redundant ends and no obvious termini, which is typical of T-even phages (21, 22). Pairwise comparisons using FastANI v1.34 revealed 79.76–98.25% whole-genome average nucleotide identity (wgANI) (23) to four RefSeq *Karamvirus* genomes that are listed in the ICTV Master Species List (https://ictv.global/msl, MSL40.v2, accessed 10 March 2026) (Fig. 1). *Karamvirus* phages are lytic, related to T4, have myovirus morphology, were isolated on species of the genera *Enterobacter*, *Klebsiella*, *Cronobacter*, *Citrobacter*, *Aeromonas*, and *Escherichia* (including MDR strains), bind to different parts of lipopolysaccharide, show stability in storage, and have relatively broad host ranges (22, 24–27; https://www.ncbi.nlm.nih.gov/Taxonomy/Browser/wwwtax.cgi?command=show&mode=tree&id=1913650&lvl=). In one study, an *Enterobacter* phage showed efficacy against *E. cloacae* infection in a zebrafish model (27).

These six *Karamvirus* phages had BACPHLIP (8) scores of 85–100%, suggesting a lytic lifestyle. There was no homology identified between their putative products and phage determinants of lysogenicity and gene transfer, or bacterial proteins, including those involved in drug resistance (12) and virulence (13). Therefore, these six phages appear to be virulent and safe for therapeutic use.

## Data Availability

Data for the phage genomes are available through NCBI under BioProject accession number PRJNA1427925. BioSample, GenBank, and NCBI Sequence Read Archive accession numbers are listed in Table 1.
